# Implication of Two Small Heat Shock Proteins in the Thermotolerance of *Bradysia odoriphaga* (Diptera: Sciaridae) Yang et Zhang

**DOI:** 10.3390/insects16111107

**Published:** 2025-10-30

**Authors:** Jiaxu Cheng, Huixin Zheng, Shuo Feng, Weiping Cao, Qingjun Wu, Jian Song

**Affiliations:** 1 Plant Protection Institute, Hebei Academy of Agriculture and Forestry Sciences, Baoding 071000, China; chengjx8023@163.com (J.C.); fengshuo9311@163.com (S.F.); cwplx751209@163.com (W.C.); 2 Key Laboratory of Integrated Pest Management on Crops in Northern Region of North China, Ministry of Agriculture and Rural Affairs, Baoding 071000, China; 3 IPM Innovation Center of Hebei Province, Baoding 071000, China; 4 International Science and Technology Joint Research Center on IPM of Hebei Province, Baoding 071000, China; 5 Key Laboratory of Green Control of Crop Pests in Hunan Higher Education, Hunan University of Humanities, Science and Technology, Loudi 417000, China; zhenghx0124@163.com; 6 State Key Laboratory of Vegetable Biobreeding, Institute of Vegetables and Flowers, Chinese Academy of Agricultural Sciences, Beijing 100081, China

**Keywords:** *Bradysia odoriphaga* Yang et Zhang, small heat shock proteins (sHsps), heat stress, gene expression, RNA interference

## Abstract

*Bradysia odoriphaga* Yang et Zhang damages roots of 30 plant species, resulting in >50% yield loss. Heat stress can not only affect the survival but also affect the expression of heat shock proteins of *B. odoriphaga*. In this study, two small heat shock protein genes, *Hsp21.9* and *Hsp22.3*, were cloned from *B. odoriphaga*. The full-length cDNA sequences of *BoHsp21.9* and *BoHsp22.3* were 749 and 941 bp in length and contained a 588 and 594 bp open reading frame (ORF), encoding a protein of 196 and 198 amino acids with a calculated molecular weight of 21.9 and 22.3 kDa and an isoelectric point of 6.84 and 6.91. Phylogenetic tree analysis showed that *BoHsp21.9* and *BoHsp22.3* clustered into one branch with flies. qRT-PCR analyses indicated that *BoHsp21.9* and *BoHsp22.3* were expressed in all tested developmental stages and body segments, especially induced by heat stress. RNAi-mediated silencing of *BoHsp21.9* and *BoHsp22.3* significantly decreased the survival rate of fourth-instar larvae when exposed to 38 °C. This is the first study on small heat shock proteins in *B. odoriphaga*, and *BoHsp21.9*, and *BoHsp22.3* play important roles in the molecular mechanism of *B. odoriphaga* to theromotolerance.

## Simple Summary

*Bradysia odoriphaga* is an important underground pest that can damage more than 30 plant species. It has been proven that *B. odoriphaga* can be killed when the temperature exceeds 40 °C for 4 h. This study identified two small heat shock protein genes, *BoHsp21.9* and *BoHsp22.3*, as essential for heat stress. The results showed that *BoHsp21.9* and *BoHsp22.3* are expressed in all developmental stages and body segments, especially expressed when induced by heat stress. RNAi-mediated silencing of *BoHsp21.9* and *BoHsp22.3* significantly decreased survival rate of fourth-instar larvae when exposed to 38 °C. This is the first study on small heat shock proteins in *B. odoriphaga*.

## 1. Introduction

*Bradysia odoriphaga* Yang et Zhang, a kind of root maggot that seriously devastates a variety of vegetables such as Chinese chive and onion, can survive in both open fields and protected cultivation [1,2,3]. The larvae cluster and destruct roots and bulb tissues, resulting in moisture loss and even death [2,3,4,5]. Among the numerous control methods of *B. odoriphaga*, a new, convenient, efficient, and environment-friendly physical method, soil solarization, has achieved great success, which is to kill *B. odoriphaga* by using plastic film to make the ground temperature reach 40 °C.

**Figure 1:**
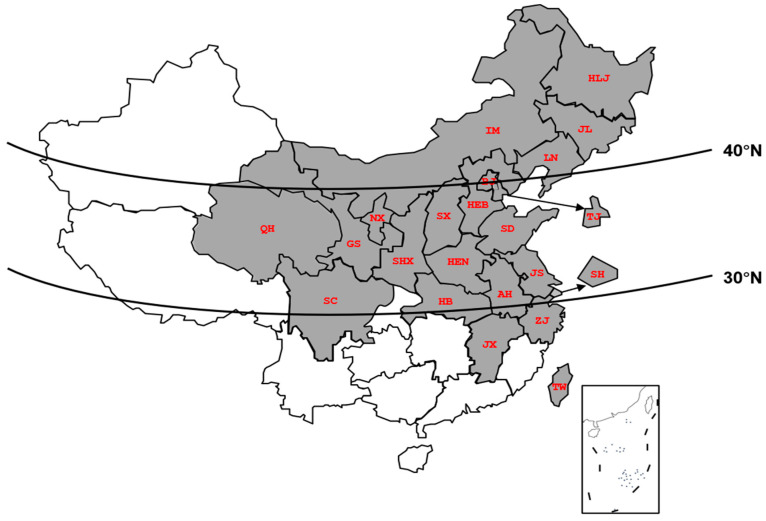
The occurrence of *B*. *odoriphaga* in China. The gray part indicates the presence of *B. odoriphaga*.

*B. odoriphaga* has two opposite characteristics of cold resistance and heat sensitivity. We found that *B. odoriphaga* is mainly distributed north of 30° N in China, where it occurs in multiple generations (Figure 1). Field dynamic monitoring showed that *B. odoriphaga* populations usually decreased in summer and winter [6]. A series of biological experiments indicated that the optimal growth temperature of *B. odoriphaga* is 20 °C to 25 °C [5]. The developmental minimum temperature threshold of *B. odoriphaga* is 7.8 °C [7] and *B. odoriphaga* could overwinter with larvae in Beijing [8]. However, the survival of *B. odoriphaga* was affected by high temperature. When temperature exceeded 30 °C, *B. odoriphaga* shortened its development duration [5]. When the temperature exceeded 37 °C for 2 h, the eggs laid by the adults of *B. odoriphaga* did not hatch [6]. When the temperature exceeded 40 °C for 4 h, all stages of *B. odoriphaga* could be killed [9]. Among all developmental stages, larvae had a lower sensitivity than other stages [9]. In addition, the transcriptome data of larvae exposed to heat stress showed that small heat shock proteins were the highest up-regulated [10].

Small heat shock proteins (sHsps) represent a diverse group of proteins with molecular weights ranging from 12 kDa to 42 kDa [11,12,13,14,15]. These proteins are ubiquitous across nearly all organisms and exhibit significant variation in their sequence, structure, size, and function [16,17]. The sHsps have a conserved α-crystallin domain which comprise about 100 amino acid residues, and variable N- and C-terminal extensions [16,18,19,20]. The α-crystallin domain forms a conserved β-sheet sandwich in sHsp secondary structure, which helps sHsps assemble into oligomeric complexes that prevent irreversible protein aggregation under extreme temperature conditions [21,22,23,24]. The expression of *Hsp27* in *Drosophila* was heat-induced in a wild temperature range of 30–37 °C, with a maximum level at 35 °C [25]. The expression of *Hsp22.6* in *Apis cerana cerana* was significantly up-regulated by temperature variations at 4 °C, 16 °C, and 42 °C [26]. In *Aedes aegypi*, *Hsp26* was up-regulated under thermal stress to protect the larvae and pupae against stressful conditions [27]. In *Bombyx mori*, the expression of *Hsp19.9*, *Hsp21.4*, *Hsp23.7*, *Hsp25.4,* and *Hsp27.4* could be induced by exposure to high temperature [22,28,29,30].

Based on field control results, field investigation results, biological experiment results, and transcriptomics results, we would like to know whether sHsps are involved in the thermotolerance of *B. odoriphaga*. Thus, in this study, we used *B. odoriphaga* larvae as materials and studied their sequence cloning, evolutionary analysis, spatiotemporal expression, and response expression to different high temperatures; and the functional study of two sHsps laid the foundation for further study on the response mechanism of *B. odoriphaga* to thermotolerance.

## 2. Materials and Methods

### 2.1. Insects

*B. odoriphaga* used in this study were the laboratory populations established in 2016 [6].

### 2.2. RNA Extraction and cDNA Synthesis

We referred to Cheng et al. for the methods used [10].

### 2.3. Cloning and Confirmation of BoHsp21.9 and BoHsp22.3

Two small heat shock proteins, one of which is partial, were identified from previous transcriptome data of *B. odoriphaga* [10]. A 3/5-RACE Kit (Clontech, Dalian, China) was used to obtain the full-length cDNA of *BoHsp21.9*. For 5′-RACE, the PCR procedures for the first round were 5 cycles of 94 °C for 30 s, 64 °C for 30 s, and 72 °C for 3 min, followed by 5 cycles of 94 °C for 30 s, 60 °C for 30 s, and 72 °C for 3 min, and followed by 25 cycles of 94 °C for 30 s, 56 °C for 30 s, and 72 °C for 3 min. The PCR conditions for the second round were 20 cycles of 94 °C for 30 s, 56 °C for 30 s, and 72 °C for 3 min.

Based on the obtained sequence of the 5′-RACE, the putative full-length of *BoHsp21.9* and *BoHsp22.3* genes were amplified from the transcriptome data using specific primers Hsp21.9-F, Hsp21.9-R, Hsp22.3-F, and Hsp22.3-R. The PCR was performed at 94 °C for 5 min, followed by 35 cycles of 94 °C for 45 s, 58 °C for 60 s, and 72 °C for 30 s, with a final extension at 72 °C for 10 min. All primers used are shown in Table 1.

### 2.4. Bioinformatic Analysis

Two full-length sHsp cDNAs were utilized to blast for homologs at the NCBI website (https://blast.ncbi.nlm.nih.gov/Blast.cgi) (accessed on 19 October 2025). Open Reading Frames (ORFs) were identified with the aid of the online ORF Finder software (http://www.ncbi.nlm.nih.gov/orffinder/) (accessed on 19 October 2025). The isoelectric point (pI) and molecular weights (KDa) of the predicted proteins were calculated by the SWISS-PROT (ExPASy server) program “Compute pI/Mw” (http://web.expasy.org/compute_pi/) (accessed on 19 October 2025). For phylogenetic analysis, twenty-one sHsps protein sequences from 10 insect species including Diptera, Lepidoptera, Hymenoptera, and Coleoptera were downloaded from GenBank. A phylogenetic tree was constructed using the MEGA 6.0 software, employing the Maximum Likelihood (ML) method with LG + G model and supported by 1000 bootstrap replicates.

### 2.5. Sampling of BoHsp21.9 and BoHsp22.3 in Different Developmental Stages and Body Segments

To create age-synchronized cohorts, approximately 10 pairs of adult *B. odoriphaga* were transferred into new Petri dishes. Fresh Chinese chive rhizomes were placed in the dishes, allowing the gnats to mate and lay eggs for 24 h. For different developmental stages expression analysis, ten developmental stages were defined as 200 eggs of two periods, E1 (24 h after oviposition), E2 (black eye), 20 entire bodies of four instars larvae (L1, L2, L3, L4), 20 pupae of two periods, P1 (white eyes), P2 (black eyes), and 20 newly emerged (<24 h) female and male adults. For the analysis of body segments expression, three body segments including head, thorax, and abdomen from 200 newly emerged female and male adults were dissected on ice. Each treatment was conducted in triplicate.

### 2.6. Sampling of BoHsp21.9 and BoHsp22.3 in Response to Heat Stress

To evaluate the expression of two sHsps in response to temperature, the fourth-instar larvae of *B. odoriphaga* were subjected to different thermal regimes. Temperature treatments were conducted in an environmental chamber (MLR-351H, Sanyo Electric Co., Ltd., Osaka, Japan), in which larvae were held in Petri dishes covered by plastic cups with holes. For this study, 30 fourth-instar larvae for each treatment were exposed to a particular temperature (30, 32, 34, 36, or 38 °C, respectively) for varying durations (1, 2, 4, 6, 8, 10, or 12 h, respectively). Temperature was controlled such that it fluctuated no more than ± 0.5 °C. After exposure, five live larvae were quickly frozen in liquid nitrogen and stored at −80 °C. And other live larvae in Petri dishes were transferred to another environmental chamber at 25 °C to allow for recovery for 1 h and 2 h. After recovery, five survival larvae were stored at −80 °C until analysis. Larvae maintained at 25 °C were used as reference control. Each treatment was conducted in triplicate.

### 2.7. Real-Time Quantitative PCR

qRT-PCR was performed on an ABI QuantStudio 3 real-time PCR system (Applied Biosystems Inc., Foster City, CA, USA) using FastFire qPCR PreMix (SYBR Green) (Tiangen, Beijing, China) with the conditions of 95 °C for 1 min, followed by 40 cycles of 95 °C for 5 s and 60 °C for 15 s. Specific primers Hsp21.9-FP, Hsp21.9-RP and Hsp22.3-FP, Hsp22.3-RP of *BoHsp21.9* and *BoHsp22.3* are shown in Table 1. The amplification efficiency (*E*) was determined by constructing a standard curve with a 3-fold cDNA serial dilution. The genes RPS15 and RPL28 were used as internal reference genes to normalize the expression levels of *BoHsp21.9* and *BoHsp22.3* among the samples, of which stability analysis under heat stress was provided by RefFiner online (http://blooge.cn/RefFinder/) (accessed on 19 October 2025) (Table S1) [31]. Three technical replications were performed for each biological replication. 2^−ΔΔCT^ methods were used to calculate the relative expression of *BoHsp21.9* and *BoHsp22.3* [32].

### 2.8. Double-Stranded (dsRNA) Synthesis

*BoHsp21.9* and *BoHsp22.3* dsRNA were synthesized using the AmpliScribe^TM^ T7-*Flash*^TM^ Transcription Kit (Lucigen Simplifying Genomics, Middleton, WI, USA). Specific dsRNA primers of *BoHsp21.9* and *BoHsp22.3* are listed in Table 1. PCR was performed with the procedures of 94 °C for 5 min, followed by 35 cycles of 94 °C for 30 s, 57 °C for 30 s, and 72 °C for 30 s, with a final extension at 72 °C for 10 min. Ethanol precipitation and nuclease-free water were utilized to purify and elute dsRNA. Double strands of green fluorescent protein (ds*GFP*) was used as the non-target negative control. Nuclease-free water was used as the negative control.

### 2.9. dsRNA Feeding

The established method by Chen et al. [33] was used to perform RNAi by feeding fourth-instar larvae dsRNA (30 µg/g artificial diet). Larvae fed with the same amount of artificial diet containing the same amount of ds*GFP* and only fed with the artificial diet were used as control, respectively. RNAi efficiency on transcript expression was analyzed using qRT-PCR at 12, 24, 48 h. The relative expression level of *BoHsp21.9* and *BoHsp22.3* were normalized with those only from the group fed with the artificial diet.

### 2.10. Heat Stress After RNA Interference

For heat stress treatments after dsRNA uptake, the exposure time (2 h and 3 h) of 38 °C was used for the survival assessment. At the time point showing the highest RNAi efficiency (48 h), each group of 30 fourth-instar larvae of *B. odoriphaga* were selected for survival estimates. The larvae were counted after a recovery period of 3 h at 25 °C to exclude those individuals that were in suspended animation. Each treatment was repeated four times.

### 2.11. Statistical Analysis

Results were shown as mean ± standard error (SE), and statistical analyses were carried out using SPSS software 19.0 (SPSS, Chicago, IL, USA). For qRT-PCR results, one-way analysis of variance (ANOVA) followed by Tukey’s multiple comparison tests was applied, and some data were transformed with log_10_ to avoid heterogeneity of variance. For survival assessment results, Kaplan–Meier followed by log-rank test was applied. Statistical differences were considered significant at *p* < 0.05.

## 3. Results

### 3.1. Cloning and Sequence Analysis of BoHsp21.9 and BoHsp22.3

**Figure 2:**
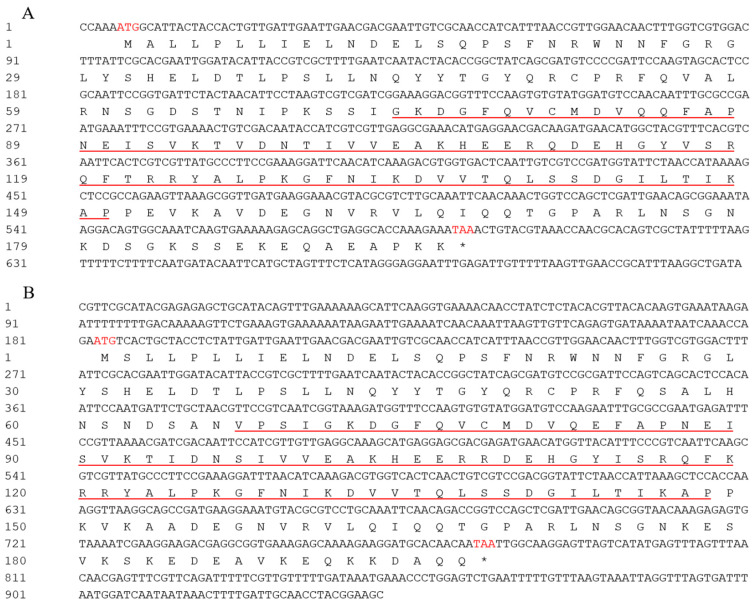
Complete cDNA sequences of *B. odoriphaga Hsp21.9* and *Hsp22.3*. (**A**) *BoHsp21.9*. (**B**) *BoHsp22.3*. The α-crystallin domain is underlined. Red font of “ATG” denote initiation codon. The asterisk and red font of “TAA” denote termination codon.

**Figure 3:**
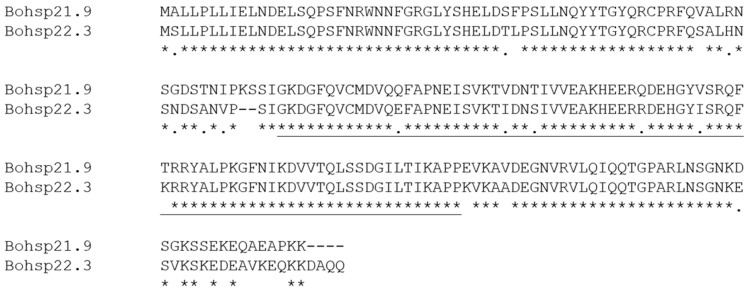
Amino acid sequence comparison of *BoHsp21.9* and *BoHsp22.3*. The asterisks and single dots denote fully and weakly conserved residues, respectively. The α-crystallin domain is underlined.

Based on previous *B. odoriphaga* transcriptome data, we identified and cloned *BoHsp21.9* (GenBase accession number: C_AA121213.1) and *BoHsp22.3* (GenBase accession number: C_AA121212.1) which might be involved in short term heat stress. *BoHsp21.9* and *BoHsp22.3* are 749 and 941 bp in length, with an open reading frame (ORF) of 588 and 594 bp that encodes 196 and 198 amino acids, respectively. The predicted isoelectric points are 6.84 and 6.91, and the calculated molecular masses of *Bohsp21.9* and *Bohsp22.3* proteins are 21.9 and 22.3 kDa, respectively. We named the genes based on their predicted molecular weights. Their deduced amino acid sequences contain the typical α-crystallin domain suggesting that they are small heat shock protein genes (Figure 2). And the amino acid sequence of *BoHsp21.9* showed 86.15% identification with *BoHsp22.3* (Figure 3).

### 3.2. Phylogenetic Analysis

**Figure 4:**
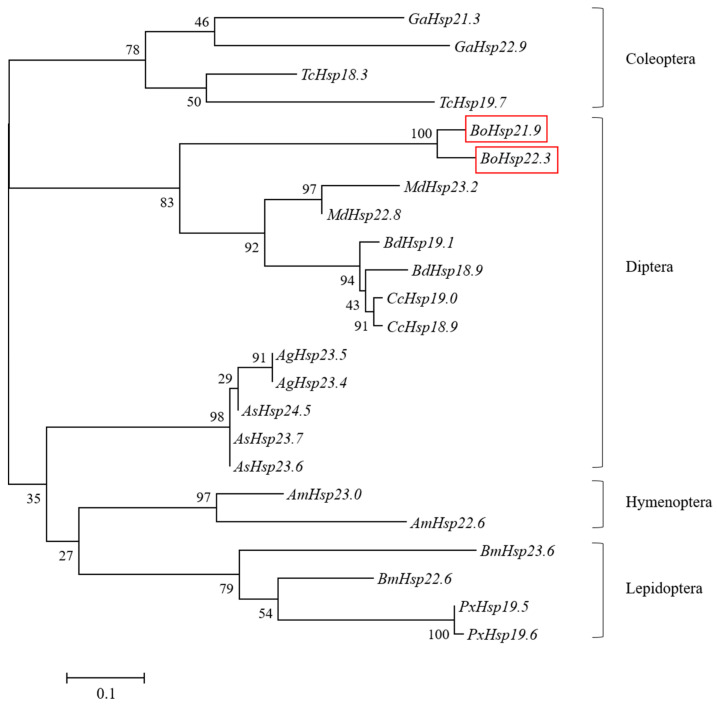
Phylogenetic analysis of *BoHsp21.9* and *BoHsp22.3*. Ten insect species full names and gene accession numbers are designated by the following abbreviations: *TcHsp19.7* (*Tribolium castaneum*, XP_973344.1), *TcHsp18.3* (*Tribolium castaneum*, XP_974367.1), *GaHsp22.9* (*Gastrophysa atrocyanea*, BAD91165.1), *GaHsp21.3* (*Gastrophysa atrocyanea*, BAD91164.1), *AmHsp23.0* (*Apis mellifera*, XP_001120194.1), *AmHsp22.6* (*Apis mellifera*, XP_001119884.1), *BmHsp23.6* (*Bombyx mori*, BAD74198.1), *BmHsp22.6* (*Bombyx mori*, ACM24354.1), *PxHsp19.5* (*Plutella xylostella*, BAE48744.1), *PxHsp19.6* (*Plutella xylostella*, AHA36865.1), *AgHsp23.5* (*Anopheles gambiae*, XP_315549.4), *AgHsp23.4* (*Anopheles gambiae*, XP_315550.4), *AsHsp24.5* (*Anopheles sinensis*, KFB40373.1), *AsHsp23.7* (*Anopheles sinensis*, KFB40371.1), *AsHsp23.6* (*Anopheles sinensis*, KFB40372.1), *MdHsp22.8* (*Musca domestica*, XP_005190092.1), *MdHsp23.2* (*Musca domestica*, XP_005190102.1), *BdHsp18.9* (*Bactrocera dorsalis*, XP_011198114.1), *BdHsp19.1* (*Bactrocera dorsalis*, XP_011198115.1), *CcHsp18.9* (*Ceratitis capitate*, ACG58884.1), and *CcHsp19.0* (*Ceratitis capitate*, XP_004523808.1).

To study the evolutionary relationships of *BoHsp21.9* and *BoHsp22.3*, twenty-one full-length sHsp amino acid sequences from Coleoptera, Hymenoptera, Diptera, and Lepidoptera were employed for phylogenetic analysis, which name and accession numbers are listed in the figure captions. As shown in Figure 4, *BoHsp21.9* and *BoHsp22.3* clustered together with other Diptera insects, and clustered with flies more nearly than midges.

### 3.3. Developmental Stage Expression Patterns of BoHsp21.9 and BoHsp22.3

**Figure 5:**
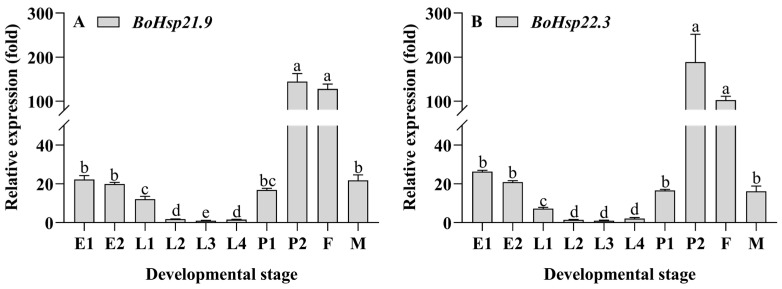
Expression patterns of *BoHsp21.9* and *BoHsp22.3* in different developmental stages. E1: 200 eggs of 24 h after oviposition. E2: 200 eggs of black eyes. L1–L4: 1st–4th instar of 20 larvae. P1: 20 pupae of white eyes. P2: 20 pupae of black eyes. F/M: 20 newly emerged female and male adults. Data are represented by means ± standard errors (Table S2). Different letters represent statistically significant differences (*p* < 0.05, Tukey HSD in one-way ANOVA).

Developmental stage expression patterns of *BoHsp21.9* and *BoHsp22.3* were measured by qRT-PCR. As shown in Figure 5 and Table S2, the expression pattern of *BoHsp21.9* and *BoHsp22.3* showed a gradual decrease from E1 stage (eggs of 24 h after oviposition) to E2 stage (eggs of black eye). During the larval stage, the expression pattern firstly decreased from L1 stage (1st-instar larvae) to L3 stage (3rd-instar larvae), followed by an increase at L4 stage (4th-instar larvae). However, the expression pattern was opposite from egg and larvae stage, with a rapid increase from P1 stage (pupae with white eyes) to P2 stage (pupae with black eyes). In addition, the expression pattern of female was much higher than that of male.

### 3.4. Body Segment Expression Patterns of BoHsp21.9 and BoHsp22.3

**Figure 6:**
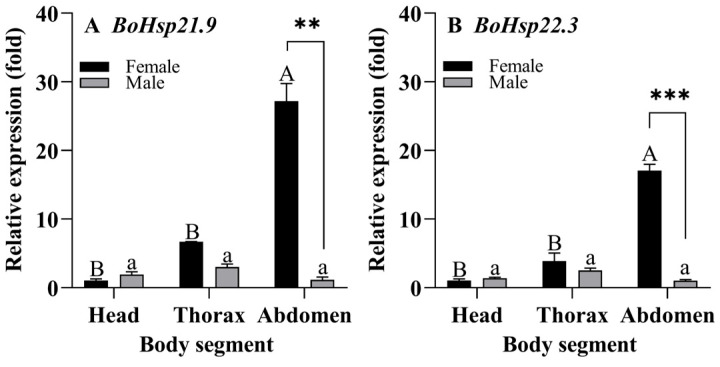
Expression patterns of *BoHsp21.9* and *BoHsp22.3* in different body segments. Head, thorax, abdomen dissected from 200 newly emerged female and male adults. Data are represented by means ± standard errors (Table S3). Different uppercase/lowercase letters represent statistically significant differences in female/male (*p* < 0.05, Tukey HSD in one-way ANOVA). “*” represents significant differences; ** *p* < 0.01, *** *p* < 0.001.

Body segment expression patterns of *BoHsp21.9* and *BoHsp22.3* were measured by qRT-PCR. As shown in Figure 6 and Table S3, the expressions of *BoHsp21.9* and *BoHsp22.3* were higher in the abdomen of female adults than in males, with 20-fold higher for *BoHsp21.9* (F_1,4_ = 95.89, *p* = 0.001) and 22-fold higher for *BoHsp22.3* (F_1,4_ = 317.081, *p* = 0.000), respectively. In addition, both genes were expressed in all parts of female and male adults, and the expression levels were lowest in the head of female adult and lowest in the abdomen of male adult.

### 3.5. Expression Patterns of BoHsp21.9 and BoHsp22.3 After Heat Stress

**Figure 7:**
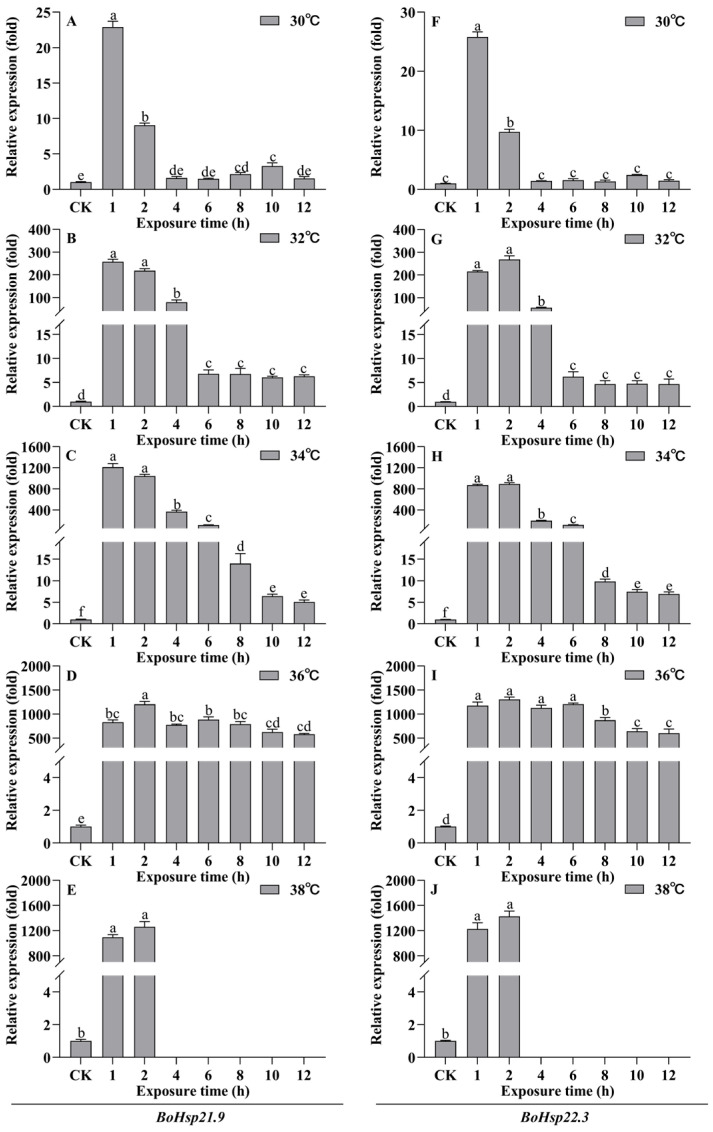
Expression patterns of *BoHsp21.9* and *BoHsp22.3* after heat stress. (**A**–**E**) represent *BoHsp21.9* at heat stress temperature of 30 °C, 32 °C, 34 °C, 36 °C, 38 °C. (**F**–**J**) represent *BoHsp22.3* at heat stress temperature of 30 °C, 32 °C, 34 °C, 36 °C, 38 °C. CK represents 25 °C. Data are represented by means ± standard errors (Tables S4 and S5). Different letters represent statistically significant differences (*p* < 0.05, Tukey HSD in one-way ANOVA).

We used heat stress to further investigate the expression of *BoHsp21.9* and *BoHsp22.3*. As shown in Figure 7, and Tables S4 and S5, *BoHsp21.9* and *BoHsp22.3* exhibited a significant upregulation in response to heat stress. The expression of *BoHsp21.9* and *BoHsp22.3* reached the maximum value at 30 °C, 32 °C, 34 °C for 1 h or 2 h. With the increase in temperature of heat stress, the maximum value of expression increased from 23-fold of 30 °C (F_7,16_ = 399.650, *p* = 0.000) to 257-fold of 32 °C (F_7,16_ = 303.093, *p* = 0.000) and 1211-fold of 34 °C (F_7,16_ = 307.010, *p* = 0.000) of *BoHsp21.9* (Figure 7A–C); 26-fold of 30 °C (F_7,16_ = 537.170, *p* = 0.000) to 268-fold of 32 °C (F_7,16_ = 335.605, *p* = 0.000) and 890-fold of 34 °C (F_7,16_ = 1186.764, *p* = 0.000) of *BoHsp22.3* (Figure 7F–H), respectively. At 30 °C, 32 °C, and 34 °C, the expression of *BoHsp21.9* and *BoHsp22.3* decreased with the increase in exposure time, and finally tended to be gentle, reaching 2-, 6-, 5-fold of *BoHsp21.9* and 1-, 5-, 7-fold of *BoHsp22.3* at normal temperature, respectively. However, the expression patterns of *BoHsp21.9* and *BoHsp22.3* under heat stress at 36 °C were different from those at 30 °C, 32 °C, and 34 °C. Specifically, the expression levels of *BoHsp21.9* (F_7,16_ = 58.495, *p* = 0.000) and *BoHsp22.3* (F_7,16_ = 59.574, *p* = 0.000) remained at a high and almost stable expression under 1–12 h exposure time (Figure 7D,I). In addition, when the heat stress temperature reached 38 °C, and the exposure time was 4 h, the larvae of *B. odoriphaga* died (Figure 7E,J).

**Figure 8:**
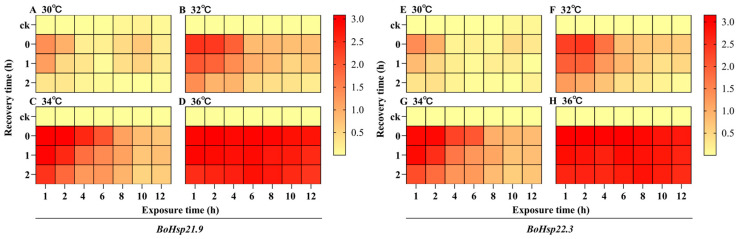
Heatmap of *BoHsp21.9* and *BoHsp22.3* expression patterns after recovery of 1 h and 2 h from heat stress. The color scale at the right ranges from the lowest (yellow) to the highest (red) relative expression fold (transformed with log_10_). CK represents 25 °C, and 0 h, 1 h, 2 h represent recovery time. Untransformed data are shown in Tables S6 and S7.

The expression patterns of *BoHsp21.9* and *BoHsp22.3* during 1 h and 2 h recovery at 25 °C from 1 to 12 h under 30–36 °C heat stress were also examined (Figure 8, Tables S6 and S7). The results showed that the expression of both *BoHsp21.9* and *BoHsp22.3* decreased significantly after 1 h or 2 h of recovery at 25 °C from 30 °C, 32 °C, 34 °C of heat stress (Figure 8A–C,E–G). The longer the exposure time, the lower the expression after recovery at 25 °C, and finally almost the same as 25 °C. However, the expression of *BoHsp21.9* and *BoHsp22.3* were significantly higher after recovery at 25 °C from 36 °C (Figure 8D,H).

### 3.6. Functional Analysis of BoHsp21.9 and BoHsp22.3 by RNAi

**Figure 9:**
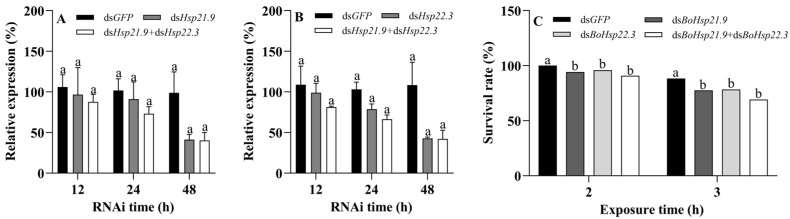
RNAi of *BoHsp21.9* and *BoHsp22.3*. (**A**) Effect of RNAi treatment on the transcript level of *BoHsp21.9* for different time points. (**B**) Effect of RNAi treatment on the transcript level of *BoHsp22.3* for different time points. The control group was fed with ds*GFP*. The transcript levels of *BoHsp21.9* and *BoHsp22.3* were examined using qRT-PCR; RPS15 and RPL28 were selected as reference genes. Data are represented by means ± standard errors (Table S8). Different letters represent statistically significant differences (*p* < 0.05, Tukey HSD in one-way ANOVA). (**C**) Survival rate of *B. odoriphaga* under 38 °C for 2 h and 3 h after 48 h RNAi. Total sample size notations: 120 4th-instar larvae (groups of 30 larvae with four replicates). Data are represented by means. Different letters represent statistically significant differences (*p* < 0.05, log-rank test).

qRT-PCR analysis showed that transcription levels of *BoHsp21.9* and *BoHsp22.3* of *B. odoriphaga* were reduced after dsRNA uptake at 12, 24, and 48 h compared with the ds*GFP* group (Figure 9A,B). After treatment with ds*BoHsp21.9* and ds*BoHsp22.3* at 48 h, *BoHsp21.9* (F_2,6_ = 4.242, *p* = 0.071) and *BoHsp22.3* (F_2,6_ = 4.749, *p* = 0.058) expression were reduced by 60% in larvae. Thus, at 48 h after the uptake of ds*BoHsp21.9* and ds*BoHsp22.3*, we evaluated the survival of dsRNA-fed *B. odoriphaga* larvae at 38 °C. Compared with the ds*GFP* group, RNAi-mediated silencing of *BoHsp21.9* obviously decreased survival rate by 5.8% (*p* = 0.0740) and 10.8% (*p* = 0.0261) when the larvae were exposed to 38 °C for 2 h and 3 h, respectively. RNAi-mediated silencing of *BoHsp22.3* obviously decreased survival rate by 4.2% (*p* = 0.0241) and 10.0% (*p* = 0.0381) when the larvae were exposed to 38 °C for 2 h and 3 h, respectively. RNAi-mediated silencing of *BoHsp21.9* and *BoHsp22.3* obviously decreased survival rate by 9.2% (*p* = 0.0007) and 19.1% (*p* = 0.0003) when the larvae were exposed to 38 °C for 2 h and 3 h, respectively (Figure 9C).

## 4. Discussion

Small heat shock proteins (sHsps) exhibit chaperone activity and reflect the response mechanism of insects to environmental extreme stress [22], which have been studied in *Drosophila melanogaster* [34], *Plutella xylostella* [35], *Mamestra brassicae* [36], *Ceratitis capitate* [37], *Sesamia nonagrioides* [38], *Liriomyza sativa* [39], and *Macrocentrus cingulum* [40]. However, the study of sHsps in *B. odoriphaga* has not been conducted. In this study, *BoHsp21.9* and *BoHsp22.3* were identified by PCR, which contained 588 and 594 bp ORFs encoding 196 and 198 amino acids, respectively. The predicted *BoHsps21.9* and *BoHsp22.3* shared high amino acid sequence similarity and grouped in same clusters with flies when analyzed by the maximum likelihood method. The significant sequence homology of sHsp indicated their evolutionary conserved and functional response to heat protection [41]. Therefore, we inferred that the function of sHsp of *B. odoriphaga* is similar to that of flies.

sHsps may play an important role in regulating development stages [42] and maintaining the normal functioning of tissues [43]. In this study, *BoHsp21.9* and *BoHsp22.3* displayed similar developmental expression patterns, with the lowest expression in larvae among all developmental stages. The phenomenon of low sHsp expression in larvae was also found in *Hsp20.5*, *Hsp20.6,* and *Hsp20.7* in *locust* [44] and *Hsp19.7* and *Hsp20.7* in *Spodoptera litura* [45]. In *B. odoriphaga*, the expression patterns of larvae developmental stages decreased from L1 to L3, followed by an increase in L4. As gregarious insects, small body size in stages L1 to L3 occupies a limited amount of space to reduce individual movement and energy consumption [46]. However, the increased expression in L4 may be due to the increasing demand for energy (i.e., fold) by maturing insects [47]. In addition, *BoHsp21.9* and *BoHsp22.3* remarkably up-regulated after the larval–pupal transformation, which is highly expressed in pupal stages. The phenomenon of up-regulation after the larval–pupal transformation can be found in *Hsp20* in *S. litura* [45]. High expression of sHsps in pupal was also found in *Hsp19.5* in *P. xylostella* [35] and *Hsp19.5*, *Hsp20.8*, and *Hsp21.7* in *L. sativa* [39]. High expression of *BoHsp21.9* and *BoHsp22.3* in pupal stages of *B. odoriphaga* suggests that they may be involved in metamorphosis. Insect metamorphosis is a process of degradation and reconstruction of tissues and organs, which may greatly induce the expression of heat shock protein genes [39,48]. On the other hand, it has been reported that the more abundant the expression level of *ApsHsp20.8* tissue, the more sensitive it is to stress [49]. The low expression level of larvae indicated that compared with other developmental states, it was the most difficult to change its survival state in the face of high temperature stress. Therefore, as long as it is lethal to larvae, it is also lethal to other development states. This is consistent with the biological experiment results of Shi et al. [9] which show that more time is needed to kill the larvae. The expression levels in female adults were found to be higher than those in male adults, indicating that female adults exhibit greater sensitivity to high temperature stress compared to male adults. Therefore, when exposed to high temperature, male adults are more likely to show heat tolerance than female adults. This is consistent with the biological experiment results of Cheng et al. [6], whereby the survival rate of male adults is higher than female adults when faced high temperatures.

The expression of *BoHsp21.9* and *BoHsp22.3* was different between male and female adults; therefore, we dissected and verified the expression in the body segments of male and female adults. There were no differences between male and female adults in head and thorax, but there were significantly differences in the abdomen. We speculate that the difference in abdominal expression between male and female adults is caused by the difference in reproductive system. It has been verified that sHsps were highly expressed in the ovary of *Tribolium castaneum*; moreover, sHsps expressed in the ovary without stress played a crucial role in maintaining normal cell development [48].

All species respond to heat shock responses by synthesizing Hsps, which is a conservative defense mechanism in acute extreme environments [50,51]. Typically, Hsps are rapidly up-regulated at the onset of stress and downregulated when favorable conditions return [52]. In addition, Hsp expression patterns may differ depending on the specific type of high temperature stress exposure [53]. In our study, we exposed the larvae to 30–38 °C for 1–12 h. The expression of *BoHsp21.9* and *BoHsp22.3* reached the highest level at 30 °C, 32 °C, and 34 °C when the larvae were exposed for 1 h or 2 h, and decreased with the increase in exposure duration. The expression of *BoHsp21.9* and *BoHsp22.3* was stable and high in different exposure times under 36 °C of heat shock. The larvae died when exposed at 38 °C for 4 h. In addition, the expression of *BoHsp21.9* and *BoHsp22.3* increased with the rise in exposure temperature. Our findings indicate that sHsp expression can be induced to varying levels depending on the degree of heat shock exposure. In sub-lethal temperature tolerance, sHsps may prevent stress-induced cytoskeletal destruction by interacting with microfilaments or stabilizing actin polymers [54,55,56]. When stress intensity exceeds the regulation capacity of the organism, denaturation of proteins can occur in the cells [57]. The expression of *BoHsp21.9* and *BoHsp22.3* recovered at 25 °C after heat shock shows that *BoHsp21.9* and *BoHsp22.3* could be accumulated. With the increase in heat shock temperature, the greater the degree of accumulation obtained, and correspondingly, more time is needed to recover to the normal level. Therefore, when the lethal temperature could not be reached, the larvae of *B. odoriphaga* could survive in large numbers and may have the ability to withstand high temperature again.

RNA interference (RNAi)-mediated gene silencing is a powerful tool for exploring gene function [58]. RNAi has been effectively employed to investigate gene function in Diptera [59,60,61]. By using oral delivery RNAi technology, we found that silencing of *BoHsp21.9* and *BoHsp22.3* significantly decreased the expression of *BoHsp21.9* and *BoHsp22.3* by 60% at 48 h after dsRNA feeding compared with the ds*GFP* group. We subsequently applied gene silencing followed by a survival assay to evaluate the role of *BoHsp21.9* and *BoHsp22.3* in temperature tolerance. The results showed the decreased survival rate of *B. odoriphaga* when exposed to 38 °C for 2 h and 3 h. Compared with the ds*GFP* group, the reduced expression level after interference made it easier for *B. odoriphaga* to change survival state under high temperature stress. Therefore, we speculate that *BoHsp21.9* and *BoHsp22.3* may be important genes involved in thermotolerance in *B. odoriphaga*.

## 5. Conclusions

In conclusion, on the basis of successful cloning of the full-length of *Hsp21.9* and *Hsp22.3* of *B. odoriphaga*, we analyzed the deduced protein sequence characteristic motifs, which indicated highly conserved structures as compared with Diptera insects of fly. Developmental stages and body segments expression patterns suggested that *BoHsp21.9* and *BoHsp22.3* might be involved in regulating development and maintaining the normal function of *B. odoriphaga*. Moreover, the expression of *BoHsp21.9* and *BoHsp22.3* can be up-regulated by heat stress, and oral delivery-mediated RNAi of *BoHsp21.9* and *BoHsp22.3* can effectively suppress the gene expression, consequently resulting in the low survival rate of *B. odoriphaga* under heat stress. These results suggest that *BoHsp21.9* and *BoHsp22.3* play an important role in thermotolerance. The results of this study have preliminarily elucidated the close relationship between small heat shock proteins and the temperature adaptability of *B. odoriphaga*, providing a theoretical basis for further exploring the temperature tolerance mechanism of this insect.

## Figures and Tables

**Table 1:** Specific primers used in this study.

Name	Primer Used	Primer Sequence (5′-3′)	Efficiency (%)	R^2^
GSP1-Hsp21.9	5′-RACE	CCTTCATCAACCGCTTTTACTTCTGGCG		
GSP2-Hsp21.9	5′-RACE	TTTGGAAGGGCATAACGGCGAGTGA		
10 × Universal Primer A Mix (UPM)	RACE	TAATACGACTCACTATAGGGCAAGCAGTGGTATCAACGCAGAGT		
10 × Universal Primer short (UPS)	RACE	CTAATACGACTCACTATAGGGC		
Hsp21.9-F	PCR	CCAAAATGGCATTACTACCAC		
Hsp21.9-R	PCR	TATCAGCCTTAAATGCGGTTC		
Hsp22.3-F	PCR	CGTTCGCATACGAGAGAGC		
Hsp22.3-R	PCR	GCTTCCGTAGGTTGCAATC		
Hsp21.9-FP	qRT-PCR	TCGTCCGATGGCATTCTAACC	93.60	0.995
Hsp21.9-RP	qRT-PCR	TTCCGCTGTTCAATCGAGCT		
Hsp22.3-FP	qRT-PCR	GTCGATCGGAAAGGACGGTT	105.13	0.990
Hsp22.3-RP	qRT-PCR	TCTTGGCGTTCCTCATGCTT		
RPS15-FP	qRT-PCR	ATCGTGGCGTCGATTTGGAT	101.03 *	0.997 *
RPS15-RP	qRT-PCR	CTCATTTGGTGGGGCTTCCT		
RPL28-FP	qRT-PCR	CGTGCCCGACATTTTCATCA	105.18 *	1.000 *
RPL28-RP	qRT-PCR	GACCAAGCCACTGTAACGGA		
Hsp21.9-RNAi FP	RNAi	TAATACGACTCACTATAGGGTTGGAACAACTTTGGTCGTG		
Hsp21.9-RNAi RP	RNAi	TAATACGACTCACTATAGGGGAAGGGCATAACGACGAGTG		
Hsp22.3-RNAi FP	RNAi	TAATACGACTCACTATAGGGAATTCAAGCGTCGTTATGCC		
Hsp22.3-RNAi RP	RNAi	TAATACGACTCACTATAGGGTTCTTTTGCTCTTTCACCGC		
dsGFP-FP	RNAi	TAATACGACTCACTATAGGCAGTGCTTCAGCCGCTAC		
dsGFP-RP	RNAi	TAATACGACTCACTATAGGGTTCACCTTGA		

* represents data obtained from Shi et al. [31].

## Data Availability

The original contributions presented in this study are included in the article/Supplementary Material. Further inquiries can be directed to the corresponding authors.

## Supplementary Materials

The following supporting information can be downloaded at https://www.mdpi.com/article/10.3390/insects16111107/s1.
